# Rehabilitation Needs of Stroke Survivors After Discharge From Hospital in India

**DOI:** 10.1016/j.apmr.2016.02.008

**Published:** 2016-09

**Authors:** Sureshkumar Kamalakannan, Murthy Gudlavalleti Venkata, Audrey Prost, Subbulakshmy Natarajan, Hira Pant, Naveen Chitalurri, Shifalika Goenka, Hannah Kuper

**Affiliations:** a International Center for Evidence in Disability, Department of Clinical Research, London School of Hygiene and Tropical Medicine, London, United Kingdom; b Institute for Global Health, Faculty of Population Health Sciences, University College London, London, United Kingdom; c T.S. Srinivasan Institute of Neurological Sciences, Voluntary Health Services Hospital, Taramani, Chennai, India; d Indian Institute of Public Health Hyderabad, Amar Cooperative Society, Madhapur, Hyderabad, Telangana, India; e Institute of Public Health–Delhi, Institutional Area, Gurgaon, Haryana, India

**Keywords:** Health services research, India, Needs assessment, Rehabilitation, Stroke, NIH, National Institutes of Health

## Abstract

**Objective:**

To assess the rehabilitation needs of stroke survivors in Chennai, India, after discharge from the hospital.

**Design:**

Mixed-methods research design.

**Setting:**

Home-based.

**Participants:**

Stroke survivors (n=50; mean age ± SD, 58.9±10.5y) and primary caregivers of these stroke survivors (n=50; mean age ± SD, 43.1±11.8y) took part in the quantitative survey. A subsample of stroke survivors (n=12), primary caregivers (n=10), and health care professionals (n=8) took part in the qualitative in-depth interviews.

**Interventions:**

Not applicable.

**Main Outcome Measure:**

Rehabilitation needs after hospital discharge.

**Results:**

About 82% of the needs expressed by stroke survivors and 92% of the needs expressed by caregivers indicated that they had a substantial need for information. The proportion of financial needs reported by the stroke survivors and the caregivers was 70% and 75%, respectively. The qualitative data revealed major gaps in access to stroke rehabilitation services. Service providers identified availability and affordability of services as key problems. Stroke survivors and their caregivers identified lack of information about stroke as major barriers to accessibility of stroke rehabilitation services. Caregivers expressed a tremendous need for support to manage family dynamics.

**Conclusions:**

The study highlights a considerable unmet need for poststroke rehabilitation services. Given the lack of rehabilitation resources in India, developing an accessible, innovative, patient-centered, culturally sensitive rehabilitation intervention is of public health importance. It is crucial for low- and middle-income countries like India to develop technology-driven stroke rehabilitation strategies to meet the growing rehabilitation needs of stroke survivors.

Stroke is the second leading cause of mortality worldwide^1^ and is associated with a wide variety of sensorimotor, cognitive perceptual, and behavioral impairments.^2^ These poststroke impairments might limit the ability of stroke survivors to independently perform their activities of daily living.^3^ Consequently, they might also restrict effective participation in family and social roles.^4^ A significant proportion of stroke survivors therefore become disabled, with profound effects on their quality of life.^5^

India, like other low- and middle-income countries, is experiencing a stroke epidemic.^6^ During the past 2 decades, the prevalence of stroke in India is estimated to range from 84 to 262 per 100,000 population in rural areas to 334 to 424 per 100,000 population in urban areas. Stroke in India therefore poses a major public health challenge, given the disabling nature of the condition and the growing magnitude of disability.

There is a dearth of information about the rehabilitation needs of persons with disabilities, especially after stroke, in India where persons with disabilities in general encounter several barriers to access rehabilitation services.^7^, ^8^, ^9^ One would expect the needs of stroke survivors in India to be substantial and diverse, given the range of disabilities caused by stroke and the existing barriers to access services.

This situation warrants an understanding of the needs of the stroke survivors living in a country like India, since this would assist in developing innovative rehabilitation interventions that are accessible, patient-centered, and culturally sensitive. It could also facilitate the efficient use of locally available resources to meet the rehabilitation needs of stroke survivors in this context. Therefore, this study was undertaken to assess the various kinds of rehabilitation needs among the stroke survivors, and the factors contributing to these needs, using a mixed-methods approach. The primary objective of this study was to assess the rehabilitation needs of stroke survivors in Chennai, India, after discharge from the hospital.

## Methods

This formative study used (1) a structured questionnaire with a purposively selected sample of 50 stroke survivors and 50 caregivers; and (2) qualitative in-depth interviews with a subsample of 12 stroke survivors, 10 primary caregivers looking after them, and 8 health care professionals involved in providing stroke rehabilitation services.

### Study setting

The study was conducted in T.S. Srinivasan Institute of Neurological Sciences–The Voluntary Health Services Multispecialty Hospital and Research Center, Chennai, Tamil Nadu, India, between August 2013 and December 2013. Formal ethics approval was obtained from Institutional Ethics committees.

### Participant inclusion and exclusion criteria

Persons were eligible for inclusion in the study if they met the following criteria: (1) they were adults; (2) they had recently received a diagnosis of stroke (within the previous 6wk) as defined by the World Health Organization^10^; (3) the stroke was of minor or moderate severity (ie, score of 1–15 according to the National Institutes of Health [NIH] Stroke Scale^11^, ^12^, ^13^); (4) they had been discharged from the hospital; and (5) they were residing at home with a primary caregiver. Stroke survivors were excluded if any of the following criteria were present: (1) severe communication problems (scoring >1 in dysarthria and best language component of the NIH Stroke Scale^11^, ^12^, ^13^); (2) severe cognitive difficulties (scoring >1 in orientation, executive function, inattention, and language components of the NIH Stroke Scale components for cognition^11^, ^12^, ^13^); (3) severe comorbidities (severe psychiatric illness, hearing loss, vision loss); (4) severe stroke (ie, scoring >15 according to the NIH Stroke Scale^11^, ^12^, ^13^); and (5) inability to provide consent autonomously.

### Quantitative methods

The survey was conducted using a structured needs assessment questionnaire, specifically developed for the study. Its purpose was to identify the rehabilitation needs of stroke survivors and the barriers and facilitators encountered by them in accessing stroke rehabilitation services. Separate questionnaire schedules were developed for stroke survivors and their primary caregivers based on the World Health Organization Disability Assessment Schedule^14^ as well as tools used in previous studies.^15^

Statistical analysis was completed using STATA 13.a The frequency of each kind of response was calculated separately, and an aggregate score was obtained for each domain. The aggregate score for each kind of response in a domain (ie, the aggregate score of “small,” “moderate,” “large,” and “very large” need) was then converted into proportions of “total needs” for each of these domains.

### Qualitative methods

Separate topic guides with open-ended questions and prompts were developed for stroke survivors, their primary caregivers, and health professionals. The in-depth interview process ended when a saturation point was reached. The purpose of the in-depth interviews was to gain a comprehensive understanding of the experiences of the stroke survivors and their primary caregivers in relation to accessing stroke rehabilitation services and their rehabilitation needs after stroke. All the interviews were audio-recorded with consent from the respondents.

The qualitative data were transcribed verbatim and translated into English. Transcribed data were then analyzed using the framework approach.^16^

## Results

### Demographics

Using hospital records, we identified 99 stroke survivors. Thirteen (13.1%) of them did not survive after hospital discharge. Twenty-one (21.2%) could not be contacted, and 15 (15.1%) resided far from the hospital. In total, 50 stroke survivors and 50 primary caregivers linked to them were selected to participate in the study. Almost all participants were living within a 20- to 30-km radius of the hospital. The demographic and clinical characteristics of the participants are shown in tables 1 and 2.

**Table 1:** Demographic and clinical characteristics of stroke survivors

Characteristics	Male Participants	Female Participants	All Participants	*P* for Male-Female Differences
Sex	33 (66)	17 (34)	50 (100)	NA
Age (y)	57.2±10.2	61.9±10.6	58.9±10.5	.13
Education: primary or higher	24 (73)	12 (70)	36 (72)	.88
Marital status: married	33 (100)	17 (100)	50 (100)	1.00
Working before stroke	30 (91)	4 (24)	34 (68)	.00∗
Currently working in the same job	6 (18)	0 (0)	6 (12)	.00∗
First-ever stroke	33 (100)	15 (88)	48 (96)	.04∗
Stroke type				
Ischemic	31 (94)	16 (94)	47 (94)	.88
Hemorrhagic	2 (6)	1 (6)	3 (6)	1.00
Stroke severity				
Minor	11 (33)	5 (29)	16 (32)	.77
Moderate	22 (67)	12 (71)	34 (68)	.77
Affected side				
Right	14 (42)	10 (59)	24 (48)	.28
Left	18 (55)	5 (30)	23 (46)	.09
Both	1 (3)	2 (11)	3 (6)	.26
Receiving physiotherapy	3 (9)	4 (24)	7 (14)	.17
Use of mobility aids	7 (21)	4 (24)	11 (22)	.87

NOTE. Values are n (%), mean ± SD, or as otherwise indicated.

Abbreviation: NA, not applicable.

∗ *P*<.05.

**Table 2:** Demographic characteristics of the primary caregivers of stroke survivors

Characteristics	Male Participants	Female Participants	All Participants	*P* for Male-Female Differences
Sex	12 (24)	38 (76)	50 (100)	NA
Age (y)	37.9±14.0	44.7±10.7	43.1±11.8	.08
Education: primary school or higher	12 (100)	28 (73.7)	40 (80)	.04∗
Employed	11 (91.6)	14 (36.8)	25 (50)	.00∗
Previous training for caregiving	0 (0)	0 (0)	0 (0)	1.00

NOTE. Values are n (%), mean ± SD, or as otherwise indicated.

Abbreviation: NA, not applicable.

∗ *P*<.05.

### Quantitative results

**Fig 1:**
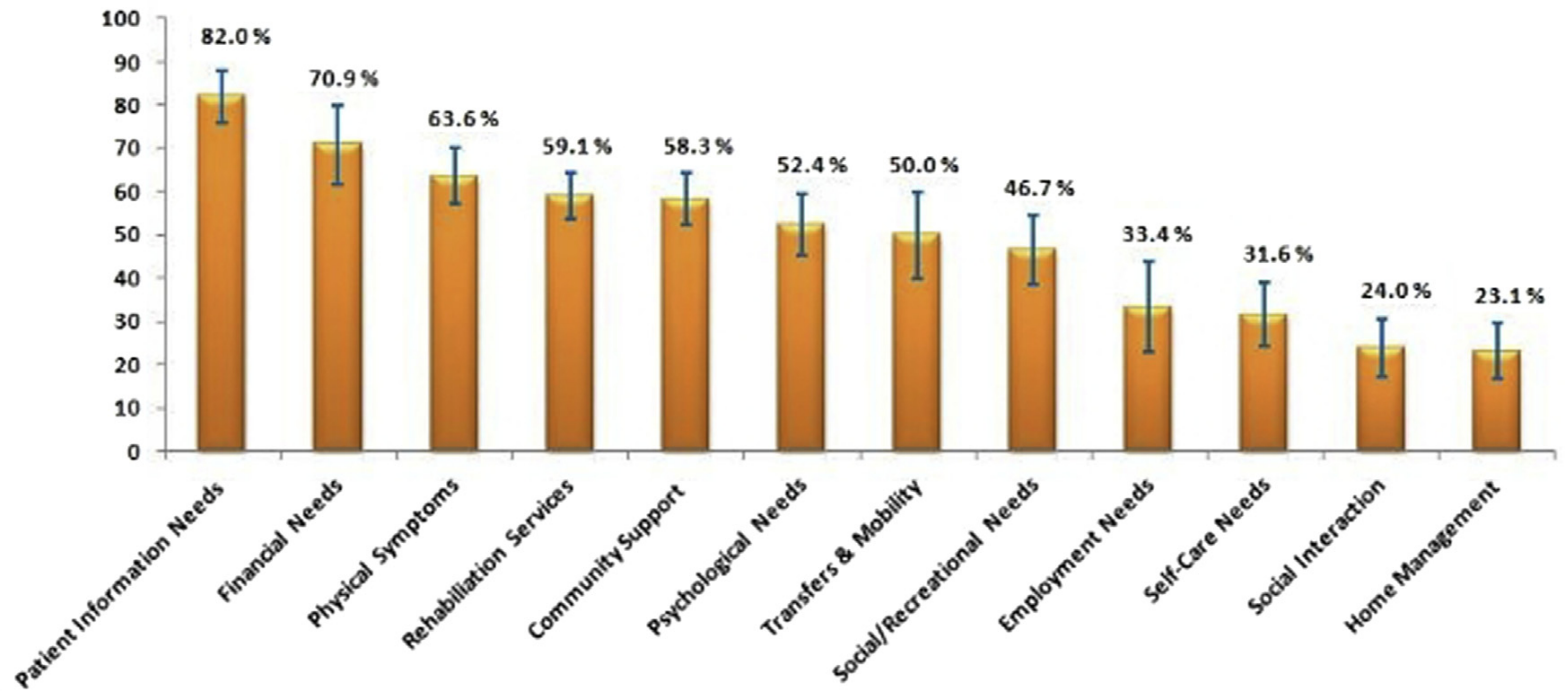
Rehabilitation needs of the stroke survivors for various functional domains as reported by the stroke survivors. Frequency of responses for various functional domains expressed in percentage.

**Fig 2:**
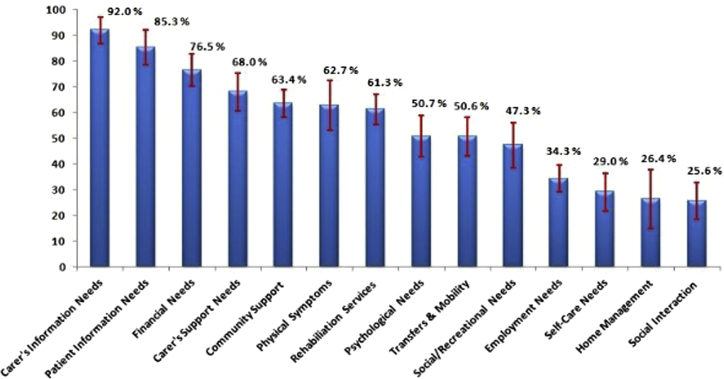
Rehabilitation needs of the stroke survivors for various functional domains as reported by the caregivers. Frequency of responses for various functional domains expressed in percentage.

All study participants reported needs in every domain incorporated in the questionnaire. None of the participants mentioned not having any rehabilitation needs. Figures 1 and 2 show the proportion of total needs for each domain reported by the stroke survivors and caregivers. The most important need for both stroke survivors and primary caregivers was related to information about “stroke and stroke rehabilitation service.” About 82% of the needs expressed by stroke survivors and about 92% of the needs expressed by caregivers in this domain indicated that they had a substantial need for information. Financial needs and support was the second most important domain for participants. The proportion of needs reported by the stroke survivors and the caregivers in this domain was nearly 70% and 75%, respectively.

The other important rehabilitation needs prioritized by both the stroke survivors and their caregivers were those related to the management of symptoms after stroke, rehabilitation services, and support in the community. The proportion of needs expressed by the stroke survivors and caregivers in these domains approximately ranged from 55% to 65%. Caregivers expressed that they need to be looked after by other family members and the community while they provided care and support to the stroke survivors. Sixty-eight percent of the responses from caregivers were related to this domain. About 50% of the needs expressed by the study participants were related to the stroke survivors' psychological needs and needs related to transfers and mobility. Both stroke survivors and their caregivers felt that stroke survivors require assistance to deal with their poststroke psychological issues and mobility problems.

The needs expressed by both the stroke survivors and caregivers for the rest of the domains were less than 50%. There was no statistically significant difference between the needs expressed by stroke survivors and their caregivers in any of these domains.

### Qualitative results

Results from the qualitative in-depth interviews agreed with and complemented findings from the quantitative survey.

#### Gaps in access to stroke rehabilitation services

**Fig 3:**
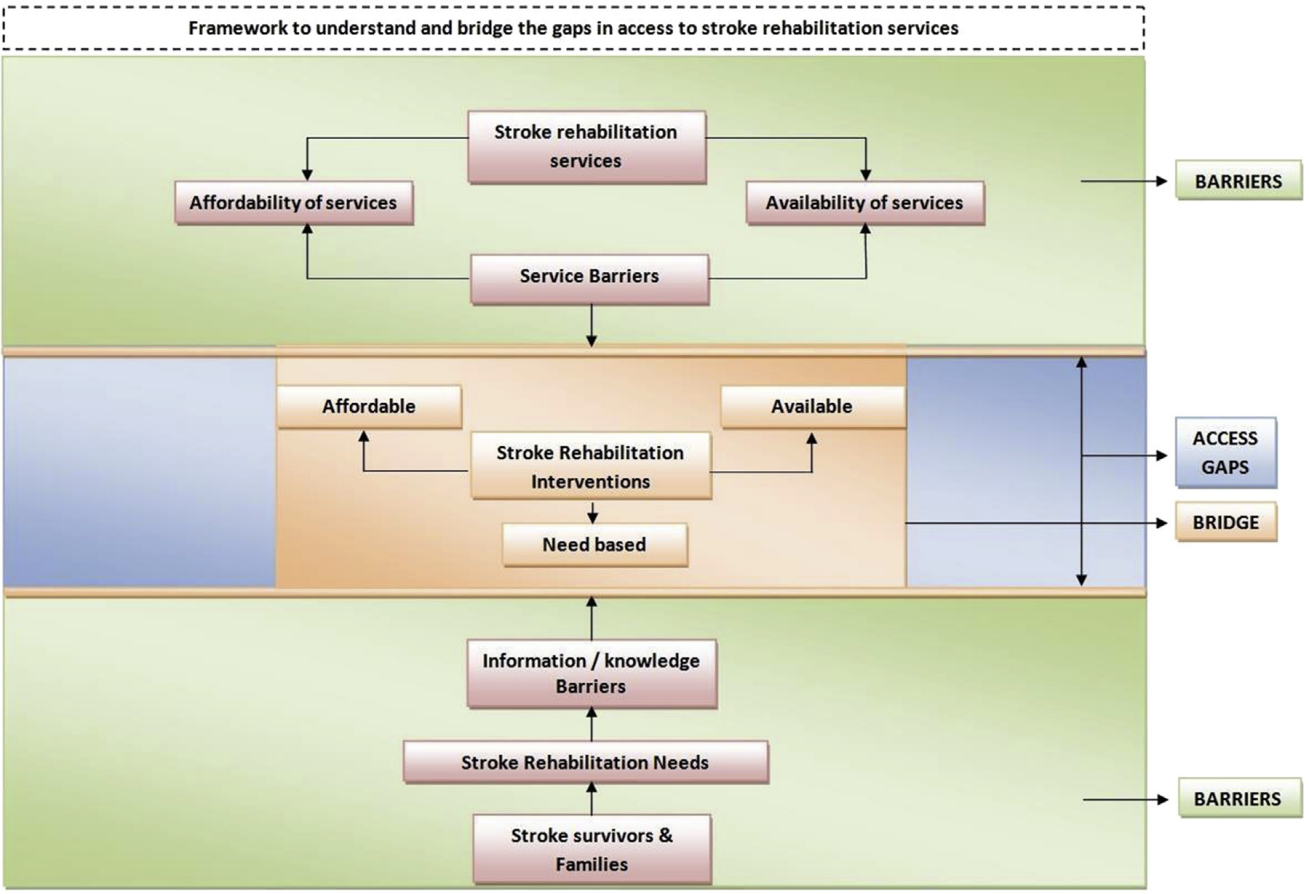
Framework to understand and bridge the gaps in access to stroke rehabilitation services.

Findings from the in-depth interviews helped investigators in deriving a framework (fig 3) for understanding the gaps in access to stroke rehabilitation services and provides reasons for the stroke survivors to have substantial rehabilitation needs. Greater details about the barriers to accessibility of stroke rehabilitation services are provided in supplemental table S1 (available online only at http://www.archives-pmr.org/).

#### Availability of rehabilitation services

There was a wide gap between the demand and supply of stroke rehabilitation services in Chennai. Findings from the study reveal that there was an acute insufficiency of rehabilitation services for people with disabilities in general, even in a major metropolitan city such as Chennai. Rehabilitation services to assist people with disabilities were hardly available to the neediest. Health providers interviewed acknowledged that there were only 2 well-known neurorehabilitation centers in the entire state of Tamil Nadu in India. An experienced physiatrist said, “The concept of rehabilitation itself is like quite new to India, I think…we are not used to… this process of rehabilitation; Here and there this has been done, but on very low scale and insignificantly.”

None of the participants reached a hospital for their stroke straight away. It took a minimum of 2 days for the respondents to find a hospital that could provide treatment and rehabilitation. Most of them reached the hospitals by word of mouth from friends and neighbors. When health professionals were asked about the efforts from the government or private health sector to address this issue, another physiatrist with expertise in evidence-based brain injury rehabilitation said, “I don't think anything substantial that's being done either in terms of primary prevention or treatment. You don't have a all in one stroke treatment and rehabilitation unit as you have in Scandinavian and European countries. So definitely, we are lagging behind in a big big way.”

Stroke survivors and caregivers reported that the quality of available services was not adequate. In general, many respondents were not satisfied with the services obtained in the hospitals where they were treated for their stroke. A health provider himself explained, “In the country, rehabilitation is almost equal to physiotherapy and physiotherapy is almost equal to passive movements of upper limbs and lower limbs. We don't have a goal-oriented, time-bound program that would aim at functional improvement.”

#### Affordability of services

There is only 1 government-managed general rehabilitation center for persons with disabilities in the entire state, and it is located in Chennai. Although rehabilitation services are free in this facility, people had to travel long distances and pay for the travel themselves to access these free services. Most often, people who could not afford to travel long distances even within the city and those who did not have the time or the money sought rehabilitation services from the nearest physiotherapy clinic. However, even this unidisciplinary therapy service was not affordable to many of the interviewed respondents. This was especially the case in poor families, when the breadwinner of the family was affected by stroke, or both. One caregiver said, “Only with his earning, our family is running. We don't have any other support and it is very difficult to be in this situation–what to do? I am clueless. I have to go for work. I should try and do any work that is available. It's just what God has in store for me.”

Given the unexpected onset of stroke, respondents said they were not prepared and often unable to organize resources for managing the problems of individuals affected by stroke within their family. Priority was given to immediate medical treatment, and most funds were spent for acute stroke treatment, which was usually expensive. Subsequently, the families ran out of funds to continue postacute rehabilitation services. An occupational therapist explained, “If a patient has a stroke, he has to take up all the …. medical expenditures on his own. When accessing a particular hospital they will be admitted in the ICU, and other medical care, for that itself they pay 1 or 2 lakhs, when it comes to rehabilitation, they may not be able to afford. Then once the money has dried out, compliance reduces and they don't complete what they started.”

Availability and affordability of stroke rehabilitation services were the major service level barriers that existed in the study context. Most people who could not afford rehabilitation services remained at home, not being appropriately looked after by family. Poststroke complications and severity of disability increase when stroke survivors do not receive appropriate rehabilitation services.^4^ Subsequently, this increases their rehabilitation needs. Given the lack of availability and affordability of stroke rehabilitation services, the rehabilitation needs of the stroke survivors were largely unmet, and the demand for available and affordable stroke rehabilitation services becomes substantial.

#### Information and knowledge barriers

Lack of information and knowledge about stroke and stroke rehabilitation services was identified as a major barrier to accessibility that existed among the stroke survivors and their family. Lack of awareness about stroke, stroke-related disability, and rehabilitation often concealed the overt demand for rehabilitation services. None of the stroke survivors, caregivers, and family members interviewed were able to identify the warning signs of stroke and seek immediate treatment for it. Most of them felt that the symptoms of stroke would resolve after rest or sleep. Most stroke survivors and the caregivers were not able to pinpoint a cause. When a stroke survivor was asked about the cause for his stroke, he said, “The doctors used to tell me frequently to check my blood pressure, but I used to tell him, ‘That and all will come and go sir.’ But now only, I am realizing that how BP affects; nobody told. I don't know that I will get stroke if I drink.”

When stroke survivors and their family were asked whether they received any information about stroke from the health care providers at hospitals where they were treated, most said that they had not.

Many stroke survivors and caregivers did not know there was a rehabilitation center located within the hospital where they received treatment for their stroke. Most also felt that the onus is on the stroke survivors and their family to gain information about the problem and on ways to manage it. A young stroke survivor expressed, “No … so far no one has given me information or given me any treatment…The situation is—Only I must do something for myself to improve.”

Health providers felt that ignorance about stroke and the inability to accept stroke-related disability among the stroke survivors and their family were major problems in communicating with them. One health provider said, “The difficulty is always in explaining the reality to the individual and family members that uh… the neurological function that is lost cannot be remediated by anymore intervention; that's the felt need for most of the patients. Nobody comes here saying that I have hemiplegia, make me walk with the quadruped; they say I am not able to use upper limb, set it right. That's the biggest challenge that we face.”

From the perspective of the service receiver, findings from the qualitative interviews suggest that lack of awareness and knowledge about stroke and the process of stroke recovery among stroke survivors and their families was an important barrier to bridging the gaps in access to stroke rehabilitation services. This was an important reason for the stroke survivors and their families to demand more information about stroke and stroke-related services (supplemental table S2, available online only at http://www.archives-pmr.org/).

#### Support for the caregivers

Support needs of caregivers came up as a major concern for the caregivers themselves and also for the stroke survivors. Caregivers and family members reported considerable change in their family roles and responsibilities when stroke occurred in a family member. Caregivers required appropriate support to physically and mentally manage these abrupt changes in roles and family dynamics (supplemental table S3, available online only at http://www.archives-pmr.org/).

## Discussion

This study identified a widespread need for rehabilitation services among stroke survivors and their caregivers in India. Information needs and financial support needs were the 2 major domains expressed by the participants. The information and support needs of caregivers were much greater compared to those of the stroke survivors. This explains the compelling need to equip caregivers as much as possible so that they can fully support the stroke survivors.

Findings from the qualitative interviews also revealed major gaps in access to stroke rehabilitation services in the study context. Overcoming barriers to the provision of stroke rehabilitation services, especially availability and affordability, appears to be essential to meet the rehabilitation needs of stroke survivors. However, the information and knowledge needs of stroke survivors and their caregivers and families should also not be underestimated while attempting to develop strategies to meet the rehabilitation needs of stroke survivors. Unless stroke survivors are informed about their need for rehabilitation and the services available for it, appropriate utilization of any kind of stroke services cannot be expected.

The demographic characteristics of the stroke survivors in this study were very similar to those in previous epidemiologic studies on stroke conducted in India.^17^ To our knowledge, this is the first needs assessment study of this sort carried out in India. Indeed, the authors were able to identify only 2 other such studies^18^, ^19^ carried out in low- and middle-income countries. These studies^18^, ^19^ also found that information was the topmost priority for the stroke survivors. Although the context is very different, findings in our study were similar to those in similar studies^15^, ^20^, ^21^, ^22^, ^23^, ^24^ conducted in high-income countries.

This study has 2 major strengths. First, it used a mixed-methods design, which enabled us to obtain a richer understanding of rehabilitation needs.^22^ Second, the assessment was not restricted to stroke survivors alone; caregivers and health care providers were also included. These 2 strategies helped us gain a better understanding of the key factors that contribute to the gaps in accessibility to stroke rehabilitation services.

### Study limitations

The study also has 2 major limitations. First, participants were all recruited from a single hospital, which limits the generalizability of our findings. Second, the sample size for the quantitative needs assessment was small, given that there was only 1 hospital that provided permission for recruitment. Similar studies in the future could involve more recruitment centers and include rural areas with poorer access to health services.

## Conclusions

Our study shows that there is a substantial unmet need for poststroke rehabilitation services in Chennai, India. Lack of awareness about stroke and of ways to manage stroke-related disabilities appears to be the primary reason for this. The financial implication of providing therapeutic care and support for stroke survivors becomes an additional burden to both stroke survivors and their families. Given the lack of resources for rehabilitation in India, developing an innovative, multidisciplinary, patient-centered, culturally sensitive rehabilitation intervention is of high public health importance. This could help bridge the gap in accessibility and potentially meet the rehabilitation needs of the stroke survivors in India. Results from this needs assessment had contributed significantly toward the development of a smartphone-enabled caregiver-supported educational intervention for management of disabilities after stroke in India. The detailed description of the intervention can be found elsewhere.^25^

## Supplier

a. STATA 13; StataCorp LP.

## Figures and Tables

**Supplemental Table S1:** Barriers to accessing stroke rehabilitation services

Contributing Factors	Mechanism by Which the Barrier Causes Inaccessibility	Coping Strategy	Policy Implications
Access to patient transportation to transfer patient to hospital and/or therapy centers	Government ambulance services are very minimal and available only for emergency purposes.	Individuals prefer to hire a taxi for transportation, or an auto rickshaw if the patient can sit, and travel to the nearest health facility.	Need for emergency/nonemergency ambulance services that would allow patients to be taken to the nearest health facility that offers stroke treatment and rehabilitation services
	Government ambulance services take patients only to the nearest government hospital, which they and their families might not prefer.		
	Private emergency ambulance services and other transport facilities are expensive.		
	Auto rickshaws might not be the preferred mode of transport if patients cannot sit.		
Access to hospitals during the acute phase of stroke	Dedicated acute stroke units do not exist in most government and private multispecialty hospitals.	Given the emergency situation, family members usually pay these charges. If they don't have sufficient funds, they will borrow money or sell some of their valuables for treatment.	Provision of acute stroke services in the nearest government primary health centers
	Intensive care units for managing acute stroke are situated only in the tertiary hospitals of major cities.		
Access to treatment during the acute phase of stroke	Admission and bed charges in intensive care units are usually expensive.	Some caregivers in the family might request the patient to be seen in the general ward itself.	Provision of government health insurance schemes that would cover the cost of acute stroke treatment
	Professional fees, specialist fees, therapy fees, and fees for using other devices such as pressure mattresses and vital monitors in the intensive care unit are usually expensive and based on the patient’s medical condition.		
Access to investigation, such as computed tomography scan and other tests	Investigation charges for computed tomography scan, magnetic resonance imaging, and special blood tests are usually expensive. Even in a government tertiary hospital where these facilities are readily available, there are charges for such investigations.	Family members usually pay these charges. If they don't have funds, they will borrow money or sell some of their valuables for treatment. Some patients and families skip the investigations and go back to their home. They only come back when they have money for the investigations.	Incorporating basic investigation and diagnostic facilities within private multispecialty hospitals Waiving the charges for investigations and diagnosis in government hospitals Streamlining the administrative processes and reducing administrative delays to ensure patient compliance
	Investigation facilities might not be available in the same hospital. Patients will often have to travel to the nearest diagnostic facility and come back to the hospital with the investigation report.		
	The administrative processes involved in getting the investigations done in a government tertiary hospital are cumbersome.		
Access to medicines and other drugs	Most of the medicines and drugs prescribed by the doctors in private hospitals are usually not available in government pharmacies.	Stroke survivors prefer to get the prescribed medicines from private pharmacies in/near the hospital paying for it. Stroke survivors with minor stroke usually manage to travel (which they pay for) to a government hospital to get these medicines for free. Relatives usually get these medicines for the patient from pharmacies.	Ensuring that the prescribed medicines for stroke treatment are available in most of the government and private pharmacies in both urban and rural pharmacies
	Individuals have to pay for the medicines prescribed by doctors in private hospitals.		Prescriptions for certain medicines/drugs can be provided by medical officers in the government primary health centers, which can then be taken to government tertiary hospital pharmacies.
	Some of the drugs prescribed are available only in pharmacies near the hospital within cities, and may not be available in rural pharmacies.		
	Some prescribed medicines are available for free from government pharmacies, but patients require a prescription from a doctor in a government tertiary hospital.		Provision of essential drugs for stroke treatment in primary health centers—making it available
	The administrative processes involved in getting the medicines from a government tertiary hospital are cumbersome.		Streamlining the administrative processes and reducing administrative delays to ensure patient compliance
	The government pharmacies are located in government tertiary hospitals in major cities. Stroke survivors have to travel in person to collect these free drugs and medicines.		Waiving the charges for drugs and medicines for stroke treatment through insurance coverage
	Individuals have to make travel arrangements and fund their travel to get these medicines.		
Access to therapy and rehabilitation services during postacute phase	Professional fees for every specialist, such as physiotherapist, occupational therapist, speech therapist, and psychologist, who meets the stroke survivors and their family.	Family members usually pay these charges. If they don't have funds, they will borrow money or sell some of their valuables for treatment. Some might request the patient to be seen in the general ward itself.	Provision of government health insurance schemes that would cover the cost of stroke rehabilitation and therapy services
	Specialist consultation fees for neurologists, therapy fees (on an hourly basis), and fees for using therapy devices such as ultrasound and electrotherapy are usually expensive.		
Access to appliances and orthotics	Orthotics and rehabilitation appliances such as wheelchairs, crutches, braces, and positioning supports are not readily available.	Most of the stroke survivors do not know about the devices and appliances that can prevent disability and promote participation. Given the cost, stroke survivors have to manage their problems/disability without these orthotics and appliances.	Increasing the availability of orthotics and appliances for stroke rehabilitation in major hospitals and pharmacies
	There are very few centers for producing these devices, and appliances are located in major cities. Hence they are usually expensive.		Developing infrastructure for manufacturing orthotics and rehabilitation appliances through government health facilities and pharmacies (in-house orthotic units)
	Therapists who are aware of the orthotic manufacturers and appliances for stroke rehabilitation usually prescribe these. Many do not.		Manufacturing different kinds of orthotics and appliances for physical rehabilitation. Not just mobility or ambulatory aids.
	Most available orthotics and appliances promote walking and mobility.		Mainstreaming the supply of orthotics and rehabilitation appliances through government health facilities and pharmacies
	Appliances for assisting a stroke survivor with everyday activities, such as brushing, bathing, or toileting, are not available.		
	There is a fee for specialists to visit the patient and take measurements to make some of these devices, or for the patient to travel to the place where these devices are manufactured.		Waiving the charges for orthotics and appliances through insurance coverage.
	Limited supplies of orthotics and appliances are available in the government rehabilitation center (which is the only center for the entire state, situated in Chennai) at affordable prices.		
	Most of these appliances are prefabricated and, hence, might not have the comfort, fit, and function specific to the needs of the stroke survivor.		Provision of guidelines for prescription of orthotics and appliances
	Assessment for fit, comfort, and function of the orthotics; advice on their appropriate use; and the wearing regimen and schedules are not usually available.		
Access to long-term therapy services after the acute stroke phase	Fully-fledged comprehensive stroke rehabilitation services are rarely available to stroke survivors. There are only 2 or 3 such centers available for the entire state.	Having paid for the hospital, therapy and treatment during the acute phase of stroke, stroke survivors and families usually run out of funds to continue therapy services for their stroke-related disabilities. Family members observe and understand some of the therapeutic exercises provided by the hospital physiotherapist during the hospital stay and become the therapists for stroke survivors.	Development and strengthening of rehabilitation systems, including manpower, infrastructure, financial allocation, policies, information systems, and supplies
	There is only 1 government rehabilitation center, situated in Chennai, for the entire state, with very minimal facilities for the provision of physical rehabilitation services in general.		
	Rehabilitation services available in hospitals and local clinics, and home visits by therapists (physiotherapy) are very expensive.		Mainstreaming the provision of rehabilitation services, along with health care services
	Stroke survivors and caregivers have to travel to the rehabilitation center every day to avail themselves of these services, which adds to the cost of treatment.		Promoting organized systems of provision of care and support for individuals affected by stroke
	Home visits by physiotherapists are based on the availability and convenience of the therapists.		Increasing the availability of government-led rehabilitation services (free). Waiving the cost of services through insurance coverage.
	Community-based rehabilitation services for persons with disability generally are not available to those most in need of these services and who cannot afford to pay for them.		
	Hospitals do not have any follow-up pathways for patients who have been treated for stroke in their hospital.		

**Supplemental Table S2:** Information needs of stroke survivors and their families

Contributing Factors	Mechanism by Which the Factors Escalate the Needs	Common Practice	Implication for Providers
1. Lack of awareness about the risk factors for stroke	Poor control and management of modifiable risk factors for stroke, including hypertension, diabetes, lack of physical activity, obesity, excessive consumption of tobacco and alcohol that can lead to the recurrence of stroke among those who have already suffered one	Affected individuals and families believe that taking the drugs prescribed by doctors will resolve problems and do not think that lifestyle modifications are required.	Provision of detailed information to affected individuals and their families about modifiable and nonmodifiable risk factors for stroke and the ways to control or manage their exposure to them
2. Lack of awareness about the warning signs of stroke	Individuals and families do not identify or recognize the warning signs of stroke, thereby delaying the process of acute stroke treatment and care, which, in turn, could worsen the brain damage caused by a stroke.	Affected individuals and families realize that a stroke has occurred only after the symptoms have worsened; before then, it was considered as general body fatigue, fever, or tiredness.	Provision of information or a mass awareness campaign to the individuals affected and their families about the warning signs of stroke to identify and initiate early treatment
3. Lack of awareness about the golden hours for treatment	There is delay in identifying the occurrence of stroke. This identification happens only when the patient is taken to a tertiary hospital. Early identification and management of stroke could limit brain damage and life-threatening situations for the stroke survivor.	Family members identify some kind of illness and weakness and take the patient to a nearest local clinic. Based on the advice from the doctor in the local clinic, the family members decide whether the individual affected should be taken to a tertiary hospital or can be managed back at home with the drugs recommended by the doctor at the clinic.	Doctors in the clinic and hospitals should be able to identify stroke based on history and examination and refer the individual affected to the nearest tertiary care hospital (if treatment cannot be provided in their hospital) as soon as possible. Since affected individuals have a tendency to go back home if they are feeling better, they should be advised about the golden hours of treatment for stroke and should be encouraged to seek treatment promptly.
4. Lack of awareness about places where appropriate stroke treatment and rehabilitation is available 5. Lack of awareness about treatment for stroke 6. Lack of awareness about rehabilitation after stroke	Based on the family’s/individual’s values, beliefs, health-seeking behavior, previous knowledge about stroke and the financial situation, a decision is made about where to seek treatment of stroke. However, the decision often leads stroke survivors to visit a minimum of 2 or 3 hospitals for treatment when they have a stroke for the first time. Stroke survivors and their families usually seek treatment at hospitals that can “cure” the problem, according to their beliefs. In doing so, their funds dry up by the time they understand where they can receive the most appropriate services. Many choose to seek nonbiomedical forms of treatment and care (eg, traditional healing, siddha) in search of a cure, rather than seeking biomedical treatment at an appropriate time. This delay in treatment and rehabilitation increases the severity of poststroke disability and the stroke survivor’s dependency on others to perform their day-to-day activities.	“Hospital shopping” is a common practice. People shop for hospitals and therapies that might have a “cure” for their stroke. Friends and family provide advice about various nonbiomedical treatments for stroke, such as siddha, Ayurveda, and traditional healing practices.	Stroke treatment and rehabilitation service providers (government and private) could inform the public or individuals affected about the importance of appropriate treatment and rehabilitation after stroke, during their acute hospital treatment and recovery, through their health care providers.
		In most rural areas, people seek the help of traditional healers. In some urban areas people also seek traditional healing, siddha, and Ayurvedic treatment, believing that these can “cure” stroke.	They could also conduct stroke awareness campaigns about their services through various communication media and create awareness about appropriate services for stroke among the public.

7. Lack of awareness about recovery after stroke	Understanding and accepting key facts about stroke and stroke-related disability is a major concern for most stroke survivors and their families. They invest money and seek various kinds of treatment to cure stroke. There is a strong expectation that the individuals affected will resume their role and routine as usual after any kind of treatment or therapy, but, when it does not happen, the stroke survivors and their families are in despair.	Many stroke survivors discontinue therapy or other treatments—even medication—and stay at home. They become dependent on other people for engaging in their previous family, social, and work roles. Some stroke survivors become completely dependent on their family, and they may or may not receive appropriate care and support. Especially because of the delayed treatment resulting from hospital shopping, the survivors’ poststroke condition tends to be poor and, therefore, the level of dependency is high. This also increases the financial burden borne by the family.	Providers could initiate community-based rehabilitation programs, even if only for patients who come to their hospital for stroke treatment. Active (stroke survivors visit hospital) and passive follow-ups (providers visit stroke survivors) of stroke survivors and their families in the form of support groups, home visits, day clinics, and *melas* (get-together) could promote better understanding about the recovery process and life after stroke among stroke survivors and their families.

**Supplemental Table S3:** Support needs of stroke survivors

Contributing Factors	Mechanism by Which the Factors Escalate the Needs	Common Practice	Implication for Providers
Change in family dynamics	Role changes and role reversals in the family are very common when a family member is affected by stroke. Sometimes, the breadwinner has to stop working and support the stroke survivor and other members of the family. Sometimes, the one who was supporting the children and the breadwinner has to start work and earn money. Sometimes, 1 person has to manage many roles, supporting the stroke survivor, family, or children and also earning money for the family.	In a nuclear family, if there is only 1 person to provide support (eg, husband or wife), this person takes over all roles and performs these as far as he/she can (role reversals and role change).	Providers can counsel the family members and caregivers and help them prepare for a change in family dynamics.
		In a joint family, other family members share various roles to support both the stroke survivors and the family (role sharing)	
Availability of the caregivers	If there is only 1 person to take over the role of the stroke survivor and also to support him/her, it becomes very difficult for that person to provide good care and support to the stroke survivor. The family usually moves into a crisis situation until other family members or friends come forward for support.	It becomes a substantial burden for the caregiver to manage various different roles effectively. Over a period of time, he/she may become depressed and physically frail.	Providers could inform the family members about the possibilities of role changes and discuss ways to effectively manage crises and family disputes.
	If more than 1 person is available to support, the women (wife, daughter) in the family usually take care of the stroke survivor. Men often assist in hospital follow-ups and also support the family financially. Absence of clarity in one’s new role leads to role clashes. For example, a woman might find it difficult to transfer an obese patient to wash them or assist in shifting them from one place to another.	Role clashes and family disputes are very common in this situation. In addition to the problems caused by stroke, affected individuals bear the additional burden of role clashes and family disputes.	
Willingness of the caregivers to engage and support	Caregivers with very little willingness, interest, or motivation to support the stroke survivor do not provide appropriate care and support. The stroke survivor might then experience various stroke-related complications.	Deformities and contractures are very common among stroke survivors who are not positioned well. Pressure sores are common if affected individuals are not mobilized at regular intervals.	Providers can empower caregivers by informing, engaging and training them in various aspects of support and care for the stroke survivor and his/her family during the period of acute stroke hospitalization.
Time constraints	Caregivers sometimes do not have time to manage their own roles, take on an additional role, and support the stroke survivor. Support to the stroke survivor becomes possible only when the caregivers are free from their usual roles.	Most often, expected support is either delayed or not provided. For example, stroke survivors might not receive their medications on time. Sometimes, the caregivers skip over the exercise sessions for the stroke survivors.	
Financial constraints	If the stroke survivor was the only earning member of the family, it becomes very difficult for caregivers to meet the financial needs of the family and meet the rehabilitation needs of the stroke survivor.	Money is borrowed or property and jewels are pledged for managing the family and supporting the treatment and rehabilitation expenses for the stroke survivor and the family.	Providers should have an appropriate therapeutic justification (clinical reason) for the services provided to the stroke survivor. Delaying the rehabilitation process and charging unreasonably for treatment should be avoided.
	If caregivers take over the role of the breadwinner, they might not be able to generate an income for the family in the same way that the previous breadwinner did.	There are many tradeoffs in the usual family expenditure in order to support the stroke survivor.	Providers can engage families and stroke survivors in setting achievable goals during their stay for treatment and rehabilitation. This might reduce the cost of therapy and treatment charges.
	Sometimes, the earning member of the family finds it difficult to continue paying for the medical and rehabilitation services for the stroke survivor.	Rehabilitation services for the stroke survivor are discontinued at some point in time.	
Values, beliefs and health seeking behaviors	Values, beliefs, and health-seeking behaviors of the caregivers related to stroke rehabilitation and treatment strongly influence the level of support given to stroke survivors. If caregivers feel that traditional practices could help the affected individual, then the physical rehabilitation for stroke is completely ignored.	Most often, stroke survivors do not have any say in the decision made about their treatment by the caregivers. They trust their family and caregiver decisions and explore various treatment options without having any clarity about the decision made.	Providers could inform and explain in detail the various options available for treatment of stroke, their purposes, and the pros and cons of these treatment options.
Caregivers' awareness about stroke	The caregivers’ awareness and understanding about stroke affect the ways in which support needs are identified and provided.	Lack of awareness among the caregivers about stroke is usually one of the most common reasons for the delay in provision of appropriate treatment and care for the stroke survivor.	Providers could educate the caregivers about stroke and the appropriate management of poststroke disability.
Knowledge about caring and supporting a stroke survivor	Lack of knowledge about the exact ways of providing care and support to a stroke survivor affect the postacute prognosis (recovery) of the stroke survivor. This results in poststroke complications such as pressure sores, tightness, contractures, and deformities of the affected side of the body.	Caregivers are ignorant about the advantages of positioning, exercises, use of pillows and devices for supporting the affected hands and legs of the stroke survivor, and engagement of the affected individual in daily tasks.	Information about basic, home-based techniques to care and support the stroke survivor could be provided to the caregivers.
Sex	Female caregivers are usually involved in supporting the stroke survivor with their personal care. But in many situations, female caregivers are dependent on a male caregiver for shifting, lifting, and transporting the stroke survivor. The routine support for personal care of the stroke survivor becomes inconsistent and not as expected by the stroke survivor.	Most stroke survivors prefer a male caregiver for mobilizing them to perform personal care tasks. Until then, they manage their personal care on the bed itself (with bedpan, urinal, sponge bath, and a setup environment kept within the reach of the stroke survivor).	Irrespective of the sex and age of the stroke survivor, providers should involve the stroke survivor, primary caregiver, and key members of the family while sharing any treatment plans and advice for the stroke survivor. Providers should be cognizant about the implications of the stroke survivor’s sex and age when helping the family make decisions about treatment and rehabilitation goals.
	Male caregivers are usually involved in helping the stroke survivor with hospital visits, therapy visits, medicine management, and home management activities. They are usually not available (being at work or managing outdoor activities) most of the times when they are required. This is when the female caregiver finds it difficult to move or mobilize the stroke survivor.	Female stroke survivors usually feel shy about seeking help from a male caregiver for their personal care tasks.	
	Female caregivers are usually less involved in decision-making about the treatment and care of stroke survivors, with male caregivers making most of the decisions. This limits the amount of information and knowledge required to make appropriate decisions for the stroke survivor in the family.	Female caregivers and stroke survivors are usually informed about the decision made by the male member of the family. Most often, they don’t raise any questions or concerns or make suggestions about the decision, especially if they are not the breadwinners of the family.	
	Female stroke survivors are not usually involved in making decisions about their treatment. They are only informed about the decision. This phenomenon is reversed for male stroke survivors. This reflects the meager amount of consideration given to the felt needs of the female stroke survivors.		
Age	In many families, care and support provided to the stroke survivors is indirectly proportional to their age. Young stroke survivors receive substantially more support because they are young and have the capacity to earn or provide support to the family in the future. More elderly stroke survivors do not receive sufficient support.	Many elderly stroke survivors are supported by a paid helper who might not know them very well, or not as well as other members of the family. Alternatively, some elderly stroke survivors are moved to old-age homes where some support services are readily available.	
